# A Wolf in Sheep’s Clothing: High-Grade Ovarian Endometrioid Carcinoma Masquerading as Pregnancy

**DOI:** 10.7759/cureus.9232

**Published:** 2020-07-16

**Authors:** Talal Almas, Muneeb Ullah, Maryam Ehtesham, Abdul Haadi, Muhammad Kashif Khan

**Affiliations:** 1 Internal Medicine, Royal College of Surgeons in Ireland, Dublin, IRL; 2 General Surgery, Maroof International Hospital, Islamabad, PAK; 3 Internal Medicine, Royal College of Surgeons In Ireland, Dublin, IRL; 4 Surgical Oncology, Federal Government Poly Clinic (Post Graduate Medical Institute), Islamabad, PAK; 5 Surgical Oncology, Maroof International Hospital, Islamabad, PAK

**Keywords:** ovarian, carcinoma, endometrioid, pregnancy

## Abstract

Ovarian carcinomas remain a cause of soaring mortality in the general population. Due to their anatomical location in the pelvis, malignant ovarian transformations often evade early detection, reaching astronomical proportions before eliciting clinically obvious symptoms. Epithelial ovarian carcinomas encompass a concoction of tumours derived from the ovarian surface epithelium, and are further subdivided into several subtypes ascertained primarily through histopathological workup. Of these subtypes, endometrioid ovarian carcinoma is noted to be a particularly well-differentiated tumour that often presents early in the disease course. Contrarily, advanced-stage tumours manifest a vague constellation of symptoms, such as abdominal distension and bloating, resulting in dilatory tumour detection. We hereby delineate an interesting case of a high-grade ovarian endometrioid carcinoma that, due to its vague presenting symptoms and a concomitant history of amenorrhea, was erroneously regarded as pregnancy. Subsequent diagnostic workup divulged a bilateral endometrioid ovarian carcinoma with associated serosal involvement and extensive lymph-vascular invasion.

## Introduction

Ovarian carcinoma remains one of the most commonly occurring gynaecological malignancies and boasts a particularly dismal prognosis [1]. Ovarian cancers can broadly be divided into epithelial cancers, germ cell tumours, and stromal tumours, reflecting the specific cell lineage implicated in the malignant transformation [2]. Of these categories, an obvious preponderance of epithelial ovarian carcinomas is noted; in the Western world, up to 90% of ovarian tumours are noted to be derived from the epithelial cells [3]. The risk factors that orchestrate the pathogenesis underlying ovarian cancers are well established, and include obesity, a positive family history, pre-existing breast, uterine or colorectal cancer, and nulliparity [4]. Due to the ovaries’ location in the pelvis, early-stage disease afflicting the ovaries rarely elicits any noticeable symptoms. Contrarily, advancing ovarian disease elicits a constellation of vague, non-specific symptoms, including abdominal swelling, bloating, early satiety, increased urinary frequency, and changes to bowel habits [5]. This means that ovarian cancers, regardless of their particular subtypes, can proliferate to exorbitant proportions before they manifest any noticeable symptoms that necessitate further diagnostic evaluation. Additionally, due to the ambiguous nature of the symptoms evoked by a growing ovarian mass, further evaluation through a plethora of physical pelvic examination, radiological imaging, tumour marker levels, and biopsy is merited. The advent of state-of-the-art diagnostic facilities has contributed towards a decreased incidence of giant ovarian tumours due to prompt detection, resulting in decreased mortality rates [5]. Imperatively, factors such as the subtype, stage, and grade of the tumour govern the eventual treatment regimen that is employed [6]. We hereby delineate the case of a 48-year-old woman who presented with massive abdominal swelling that was erroneously ascribed to pregnancy. Further diagnostic evaluation divulged a high-grade endometrioid ovarian carcinoma, a subtype of epithelial carcinoma characterized by solid, cystic masses.

## Case presentation

**Figure 1:**
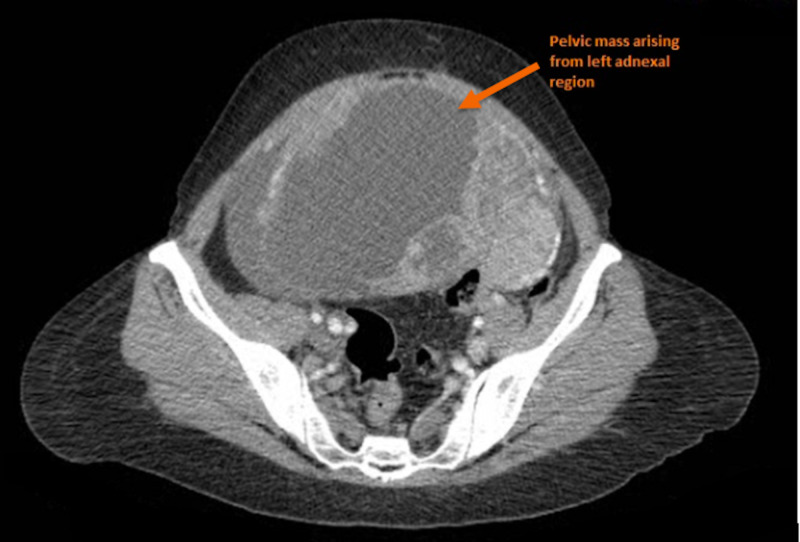
A depiction of the abnormal mass (arrow) arising from the left adnexal region.

We elucidate the case of a 48-year-old female who presented to our department with a 10-month history of persistent lower abdominal pain, massive abdominal swelling, and intermittent nausea. This constellation of symptoms was on a background of amenorrhea for the past three months, alluding to the possibility of a pregnancy. Due to her limited financial means and an inability to access adept healthcare, the patient had initially consulted a midwife, who, upon a cursory physical examination, attributed the patient’s symptoms to an uncomplicated pregnancy. In the aftermath of the consultation, however, the patient started experiencing urinary frequency, weight loss, and malaise, raising suspicion for a more sinister underlying aetiology. The patient then consulted a physician; a prompt diagnostic evaluation, through a concoction of radiological imaging and blood workup, was then commenced. Subsequent CT imaging revealed a large cystic mass in the left adnexal region, with the ovaries boring into the mass and thus indistinguishable from the abnormal growth (Figure 1). 

**Figure 2:**
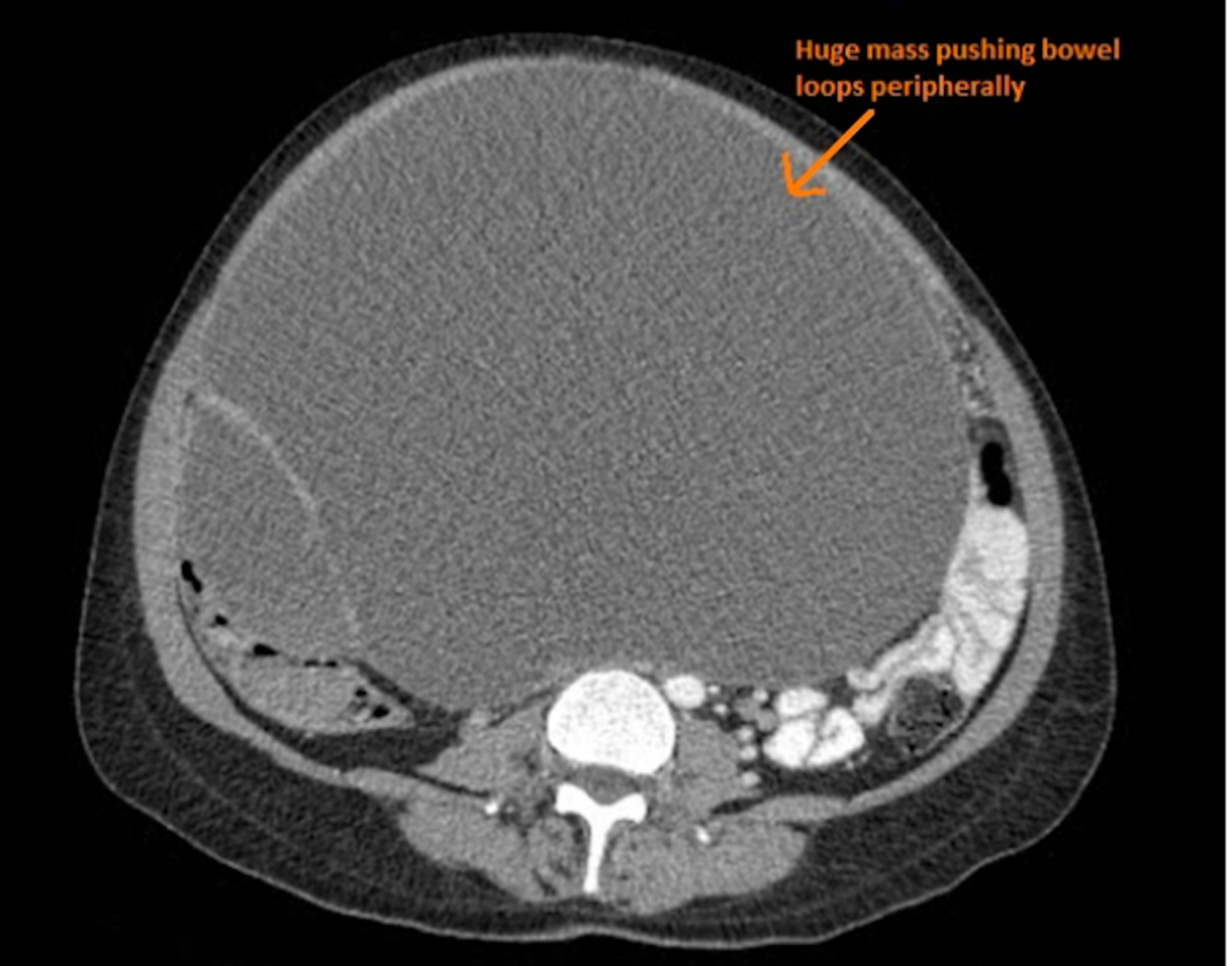
An elucidation of a giant mass (arrow) displacing the bowel loops peripherally and impinging on the urinary bladder.

Additionally, the CT scan divulged a huge mass displacing the small bowel loops peripherally and impinging on the urinary bladder (Figure 2). 

**Figure 3:**
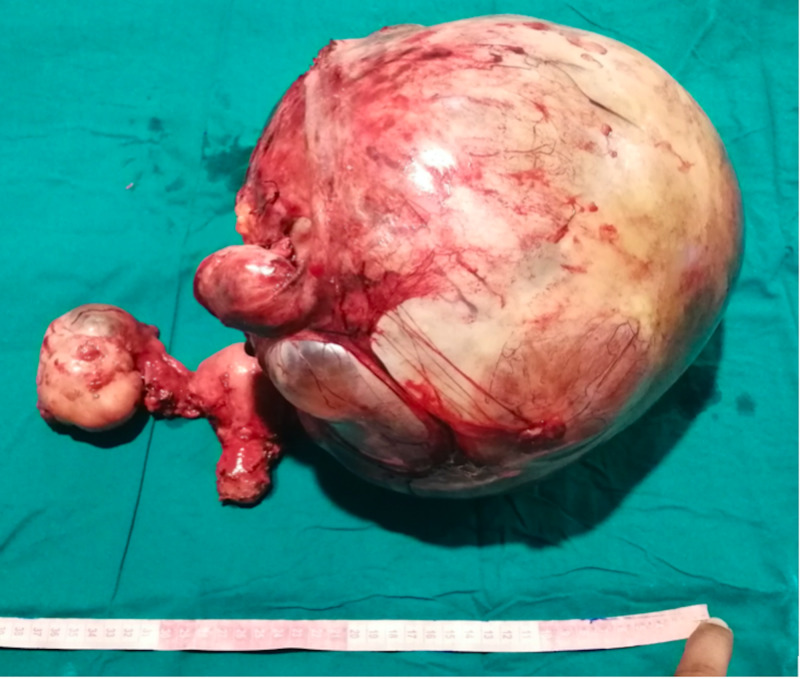
The gross morphology of the excised specimen.

The radiological findings, coupled with the patient’s presenting history, significantly raised the possibility of a malignancy. Thereafter, further diagnostic evaluation through the means of a tumour-marker profile was conducted. The radiological findings, along with the patient’s history, were discussed in a multidisciplinary team meeting (MDT); a unanimous consensus vouching for a surgical operation was reached. Preoperatively, two blood transfusions were performed in order to bolster the low blood haemoglobin levels prior to surgery. A lower midline laparotomy and a total abdominal hysterectomy with bilateral salpingo-oophorectomy were performed, yielding an exorbitant mass weighing 12 kilograms (Figure 3). 

The postoperative recovery was uneventful, and the patient was discharged on the fifth day postoperatively. The mass obtained from surgery was subsequently sent for histopathological evaluation. Gross examination revealed a tumour afflicting both the left and the right ovaries, measuring 21 x 21 x 13 cm and 8.5 x 6.0 x 5.5 cm, respectively. Additionally, both fallopian tubes were also positive for carcinoma. Histopathological workup further revealed a high-grade endometrioid ovarian carcinoma afflicting both the ovaries with concomitant serosal involvement and positive ascitic fluid. Sections obtained from both the ovarian masses showed a glandular configuration of a highly malignant tumour with a confluence of glands, papillary configurations, and areas of solid nests. A significant nuclear stratification, increased mitoses, apoptoses, and areas of necrosis and rare foci of squamous differentiation were also appreciated. Imperatively, no psammoma bodies were identified. Furthermore, lymph-vascular invasion was identified. Taken together, these histopathological findings insinuated an unequivocal diagnosis of a high-grade ovarian endometrioid carcinoma. Immunohistochemical staining was further performed with adequate controls. 

## Discussion

Ovarian cancer remains an exceedingly commonly occurring malignancy amongst women, being the eighth most common cause of cancer-related mortality, annually affecting 240,000 women and causing 150,000 deaths around the world [7]. The risk of ovarian cancer increases with age, with women in their seventies constituting the most at-risk population. While germ cell tumours are noted to be the subtype that most frequently afflicts women before the age of 40 years, epithelial tumours demonstrate a particular preponderance in women older than 40 years [8]. Imperatively, the five-year survival rate for women with ovarian cancer hovers around 45%, representing a lower survival rate than that of breast, endometrial, and cervical cancers [8]. The advent of state-of-the-art diagnostic modalities has contributed towards increasingly prompt cancer detection, significantly ameliorating cancer-related mortality rates. Despite these advances, a vast majority of ovarian cancers circumvent prompt detection, often enlarging to exorbitant dimensions before eliciting clinically evident symptoms. Further confounding their prompt detection is the constellation of vague symptoms, such as abdominal distention, bloating, and urinary urgency, that an advanced ovarian malignancy elicits. Additionally, the lack of an effective screening program, coupled with the aforementioned confounding variables, results in advanced disease being diagnosed in approximately 70% of those affected [9].

The pathogenesis of epithelial ovarian tumours has remained at the epicentre of an oncological conundrum amongst researchers. Due to their uncertain origins, ovarian cancers are currently believed to arise de novo [10]. It is presently understood that mutations involving the BRCA1, BRCA2, and tp53 can herald the onset of the histopathological derangements that eventually culminate in a malignancy [11]. Interestingly, research has also alluded to a potential correlation between certain mutations and the grade of the associated ovarian carcinomas. For instance, mutations involving KRAS have been associated with low-grade serous carcinomas [11]. On the other hand, mutations afflicting TP53 are associated with high-grade ovarian carcinomas, thus portending a worse prognosis [11].

The clinical presentation of ovarian cancer closely mimics that of a myriad of other malignancies. Most notably, stark similarities amongst the presentation of ovarian, breast, and gastrointestinal cancers are noted, deluding the timely diagnosis of an ovarian carcinoma. Furthermore, non-specific symptoms such as vague, poorly localized abdominal pain are often incorrectly ascribed to unrelated aetiologies, including endometriosis and diverticular disease [12]. Although exceedingly rare, symptoms elicited by an ovarian tumour, such as abdominal bloating, can masquerade as pregnancy [13]. In our case, a history of vague symptoms, coupled with the presence of amenorrhoea for three months, culminated in the midwife erroneously attributing the patient’s symptoms to pregnancy. Furthermore, the patient’s limited access to appropriate healthcare and diagnostic facilities rendered reaching the true underlying diagnosis a dilatory process. Thus, the patient presented with an advanced-stage ovarian tumour that warranted surgical excision. Due to extensive serosal involvement and lymph-vascular invasion, the surgical excision manifested a considerable surgical challenge; a MDT therefore remains pivotal in determining the most appropriate surgical approach. 

## Conclusions

Ovarian carcinomas remain a source of significant cancer-related mortality today. Despite the advent of modern diagnostic facilities, the vague symptoms elicited by enlarging ovarian masses can obscure their definitive diagnosis. Ovarian cancers therefore frequently evade prompt detection, manifesting noticeable symptoms only upon reaching exorbitant proportions. Furthermore, the list of differential diagnoses pertaining to amenorrhea should be broadened to include a possible underlying ovarian malignancy. Meticulous diagnostic evaluation through the means of radiological imaging, clinical examination, and blood workup remains imperative in ascertaining the underlying aetiology. 
